# Supplementary material for: J-aggregates of meso-[2.2]paracyclophanyl-BODIPY dye for NIR-II imaging
Source: Nat Commun. 2021 Apr 22;12:2376. doi: 10.1038/s41467-021-22686-z (PMC8062432; doi:10.1038/s41467-021-22686-z)
Supplement: Supplementary file 1 — Supplementary Information [file 41467_2021_22686_MOESM1_ESM.pdf]

Supporting Information

for

J-Aggregates of *meso*-[2.2]Paracyclophanyl-BODIPY Dye for NIR-II Imaging

Kang Li,[#a] Xingchen Duan,[#b] ZhiyongJiang,[a] Dan Ding,[b] Yuncong Chen,*[c] Guo-Qiang Zhang,*[b] Zhipeng Liu*[a]

[a]College of Materials Science and Engineering, Nanjing Forestry University, Nanjing 210037, China. Email: zpliu@njfu.edu.cn.
[b]Key Laboratory of Bioactive Materials, Ministry of Education, and College of Life Sciences, Nankai University, Tianjin 300071, China. Email: guoqiangzhang@mail.nankai.edu.cn
[c]State Key Laboratory of Coordination Chemistry, School of Chemistry and Chemical Engineering, Nanjing University, Nanjing 210093, China. Email: chenyc@nju.edu.cn.
# These authors contributed equally to this work.

Table of Contents

1. Methods and materials **S2**
2. Synthesis **S2**
3. X-ray crystallographic analysis **S3**
4. Photophysical properties **S3**
5. Theoretical modeling **S3**
6. Supplementary Tables and Figures **S4**
7. Coordinates of optimized structures **S18**
8. References **S31**

1. Methods and materials

All chemicals were commercially available and used as received without further purification unless otherwise specified. All reactions of air-sensitive compounds were carried out under dry nitrogen by using Schlenk techniques. Reaction progress was monitored by thin-layer chromatography (TLC) on silica plates (250 μmol/L thickness, bought from Qingdao Haiyang Chemical Co.) and spots were visualized by UV254 fluorescent indicator. Flash column chromatography was carried out using silica gel (200–300 mesh) bought from Qingdao Haiyang Chemical Co. The ^{1}H and ^{13}C NMR spectra were acquired over a Bruker AV-600 and AVANCE III HD spectrometer. HRMS spectra were recorded on a NeXion 300X spectrometer. A transmission electron microscope (TEM, JEM-1400, Japan) was used to investigate the structural features and the morphological of the samples.

2. Synthesis

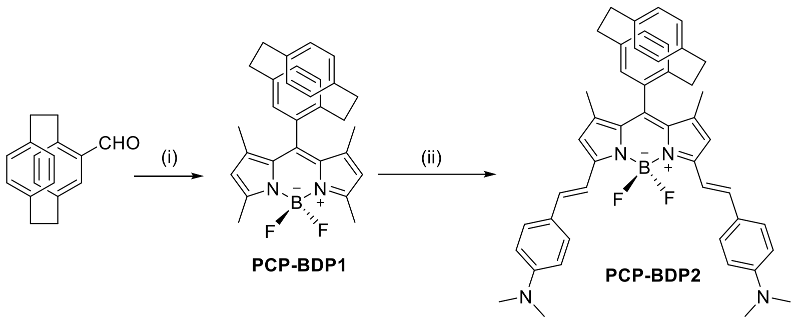

Supplementary Figure 1. Synthetic routes for PCP-BDP1 and PCP-BDP2. Reaction conditions: (i) 2,4-dimethylpyrrole (2.0 equiv), TFA (0.1 equiv), DDQ (1 equiv), Et_3N (14 equiv), $BF_3 \cdot Et_2O$ (15 equiv), DCM, r.t. 6 h; (ii) 4-dimethylaminobenzaldehyde (2.2 equiv), piperidine (10 equiv), glacial acetic acid (13 equiv), molecular sieves (4 Å), toluene, reflux, 38 h.

Ph-BDP1 and **Ph-BDP2** were synthesized according to the literature.[1]

Synthsis of PCP-BDP1. To a mixture of 4-formyl[2.2]paracyclophane[2] (472 mg, 2.0 mmol) and 2,4-dimethylpyrrole (268 mg, 4.0 mmol) in CH_2Cl_2 (50 mL) was added trifluoroacetic acid (20 μL). After the reaction mixture was stirred at room temperature for 3 h, 2,3-dichloro-5,6-dicyano-1,4-benzoquinone (DDQ) (454 mg, 2.0 mmol) in CH_2Cl_2 (20 mL) was added at 0 °C. Then the mixture was warmed up to room temperature and stirred for additional 1 h. Et_3N (3.9 mL, 28.0 mmol) and $BF_3 \cdot Et_2O$ (3.8 mL, 30.0 mmol) were added, and further stirred at room temperature for 2 h. The reaction mixture was washed with water (3×100 mL) and the organic layers were combined, dried over anhydrous $MgSO_4$ and evaporated in vacuo. The crude product was further purified using column chromatography to give **PCP-BDP1** as an orange power (136 mg, 15%). TLC (EtOAc: hexane, 1: 15 *v*/*v*): R_f = 0.3. ^{1}H NMR (500 MHz, $CDCl_3$) δ (ppm), 6.88 (d, *J* = 7.7 Hz, 1H), 6.82 (s, 2H), 6.21 (d, *J* = 9.8 Hz, 2H), 5.83 (s, 1H), 3.50–3.32 (m, 2H), 3.11–2.80 (m, 6H), 2.77 (d, *J* = 6.8 Hz, 3H), 2.61 (s, 3H), 2.50 (s, 3H), 0.77 (s, 3H). ^{13}C NMR (100 MHz, $CDCl_3$) δ (ppm), 154.5, 144.1, 143.0, 140.0, 140.0, 139.3, 137.0, 136.3, 134.1, 132.6, 132.4, 131.5, 131.1, 130.6, 126.5, 121.8, 121.4, 109.9, 77.2, 76.9, 76.66, 35.2, 34.9, 34.9, 33.0, 17.0, 14.6, 14.6, 13.7. HRMS (m/z): $[M+H]^+$ calcd. for $C_{29}H_{30}BF_2N_2^+$, 455.2465; found, 455.2451.

Synthsis of PCP-BDP2. PCP-BDP1 (140 mg, 0.31 mmol) and 4-dimethylaminobenzaldehyde (120 mg, 0.68 mmol) were refluxed for 38 h in a mixture of toluene (20 mL), piperidine (0.28 mL, 3.10 mmol) and glacial acetic acid (0.23 mL, 4.03 mmol) together with a small amount of molecular

sieves (4Å). After cooling to room temperature the mixture was placed on top of a silica column and eluted with CH_2Cl_2/hexane (1: 1, v/v) to give PCP-BDP2 as a dark green powder (81 mg, 37 %). TLC (CH_2Cl_2: hexane, 1: 1 *v/v*): $R_f = 0.3$. ^{1}H NMR (600 MHz, $CDCl_3$) δ (ppm), 7.71 (d, J = 16.1 Hz, 1H), 7.62 – 7.55 (m, 3H), 7.53 (d, J = 8.7 Hz, 2H), 7.15 (d, J = 16.1 Hz, 1H), 6.93 – 6.88 (m, 2H), 6.87 – 6.81 (m, 2H), 6.74 (dd, J = 21.2, 8.8 Hz, 4H), 6.53 (d, J = 7.7 Hz, 1H), 6.48 (s, 1H), 6.40 (s, 2H), 6.29 – 6.24 (m, 1H), 3.49 – 3.36 (m, 2H), 3.09 – 2.83 (m, 20H). ^{13}C NMR (150 MHz, $CDCl_3$) δ 152.3, 152.1, 150.8, 142.0, 140.5, 140.2, 139.3, 139.1, 138.6, 137.3, 136.9, 136.3, 136.2, 136.1, 133.8, 133.1, 132.6, 132.4, 131.5, 131.3, 131.2, 129.0, 127.3, 125.4, 125.2, 118.0, 117.5, 115.3, 115.3, 115.1, 115.0, 112.1, 112.1, 77.2, 77.0, 76.8, 40.3, 40.2, 35.2, 35.0, 33.0, 31.9, 31.4, 30.3, 30.2, 29.7, 29.3, 22.7, 17.3, 14.1, 13.7. HRMS (m/z): $[M+H]^+$ calcd. for $C_{47}H_{48}BF_2N_4^+$, 717.3935; found, 717.3901.

3. X-ray crystallographic analysis

Red single crystals of PCP-BDP1 and PCP-BDP2 were crystallized from a solution of CH_2Cl_2. Intensity data of PCP-BDP1 and PCP-BDP2 were collected on a Bruker SMART APEX-II CCD X-ray diffractometer with Cu-$K\alpha$ radiation (λ = 1.54184 Å). The structure was interpreted and refined by SHELX-2014.[3] All the ball and sticks structures of were drawn through CYLview 20.[4]

All non-hydrogen atoms were refined with anisotropic displacement parameters. Hydrogen atoms bonded to carbon were placed in geometrically idealized positions and refined using a riding model with C–H = 0.93 (aromatic) and 0.96 Å (methyl), and the isotropic displacement parameters were assigned Uiso(H) = 1.5Ueq (C) for methyl group and Uiso(H) = 1.2Ueq(C) for other H atoms. The atoms C24, C25, C26, C27, C28, C29, N4, C46 and C47 were disordered over two orientations, with refined site occupation factors of 0.523(7): 0.477(7). The ADPs of C9, C10, C11, C12, C13, C14, C15, C16, C17, C18, C39, C19, C20, C21, C22, C23, C30, C31, C32, C33, C34, C35, C36, C37, C38, C41, C42, C43, C44, C45, B1, F1, F2, N1 and N2 were restrained to be isotropic within a standard deviation of 0.02 $Å^2$. And the bond lengths of N1-C2, N1-C1, N2-C37, N3-C1, N3-C2 and N2-C30 were restrained to 1.496(13) and 1.506(13) Å within a standard deviation of 0.01 Å. Total 1544 restraints were used to model the disordered groups, and which result in the alerts B of the structure.

4. Photophysical properties

UV/vis absorption spectra were measured at room temperature on a Shimadzu UV-1750 spectrophotometer with a resolution of 1.0 nm, using quartz cuvettes of 1 cm path length. Fluorescent spectra of PCP-BDP1, Ph-BDP1, and Ph-BDP2 were recorded on a FLUOROMAX-4 spectrometer under an air atmosphere at room temperature. Fluorescence spectra of PCP-BDP2 in THF-water binary solvents and the crystalline powder state were measured on Edinburgh FLS1000 by exciting with an 808 nm laser. Fluorescent spectra of PCP-BDP2 NPs were measured on HORIBA FL-3 by exciting with an 808 nm laser.

5. Theoretical modeling

TD-DFT calculations were performed at the density functional theory level (B3LYP) with the 6-31G(d) basis set for C, H, N, B, and F atoms, using the Gaussian 09 software package.[5] The polarized continuum model (PCM) was used to provide a DCM solvation environment.

6. Supplementary Tables and Figures

Supplementary Table 1 Chemical structure and photophysical properties of reported BODIPY J-aggregates

**ref**	**Compound**	$\lambda_{abs}/\lambda_{em}$
42	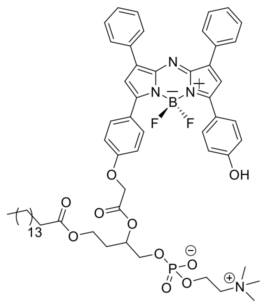	670 nm/720 nm in PBS buffer. ACQ
43	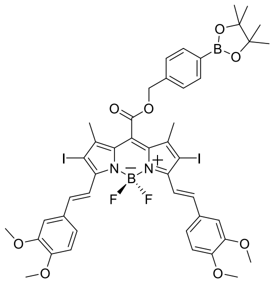	788 nm/NA in DMSO/H_2O (1/90, v/v) ACQ
46	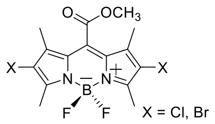	619 nm/621 nm in CH_3CN/H_2O (0.1/99.9, v/v)
This work	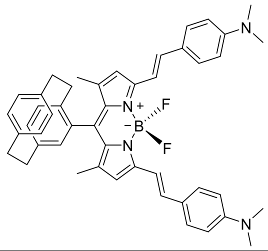	750 nm/1010 nm in PBS buffer.

Supplementary Table 2 Crystal data

Compound	PCP-BDP1	PCP-BDP2
Empirical formula	$C_{29}H_{29}BF_2N_2$	$C_{47}H_{48}BF_2N_4$
Formula weight	454.35	717.70
Crystal system;	monoclinic	triclinic
space group	P121/n1	P1
Unit cell dimensions	a = 7.257 (2) Å<br>α = 90°<br>b = 15.405 (3) Å<br>β = 93.57 (2)°<br>c = 20.881 (4) Å<br>γ = 90°	a = 8.9866 (11) Å<br>α = 91.869 (7) °<br>b = 9.3762 (10) Å<br>β = 98.726 (9)°<br>c = 26.506 (3) Å<br>γ = 90.267 (7) °
Volume	2330.0 (10) $Å^3$	2206.3 (4) $Å^3$
Z;	4	2
Cal. density	1.295 mg/m^{-3}	1.080 mg/m^{-3}
F（000）	960	762
Crystal size	0.08 x 0.08 x 0.07 mm^3	0.1 x 0.05 x 0.05 mm^3
GOF	0.977	0.960
R indices	R_1 = 0.0737，<br>wR_2 = 0.1568	R_1 = 0.1383，<br>wR_2 = 0.3147

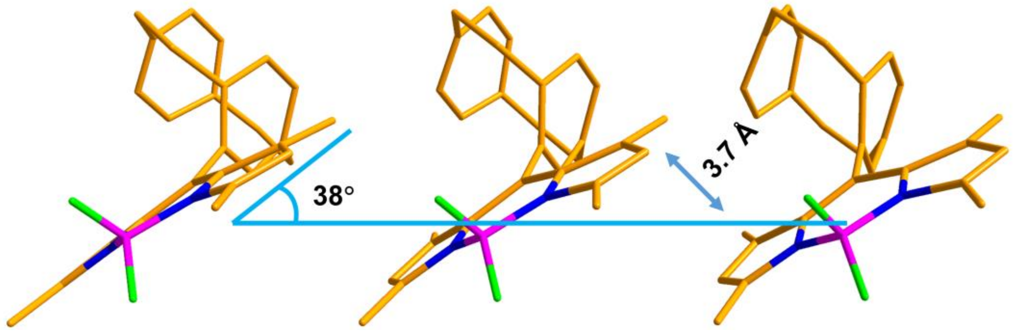

Supplementary Figure 2. Packing diagram of PCP-BDP1. Solvent molecules and H atoms are omitted for clarity.

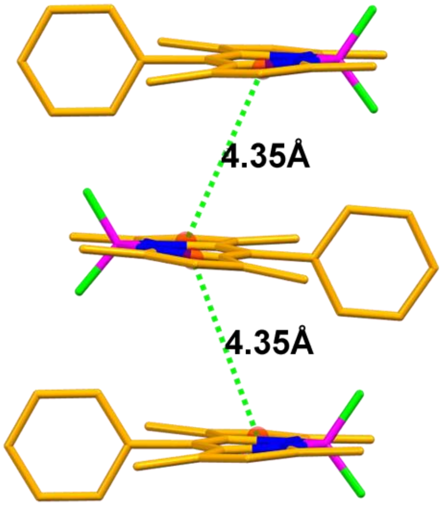

Supplementary Figure 3. Packing diagram of Ph-BDP1 (CCDC No. 1858006). H atoms are omitted for clarity.

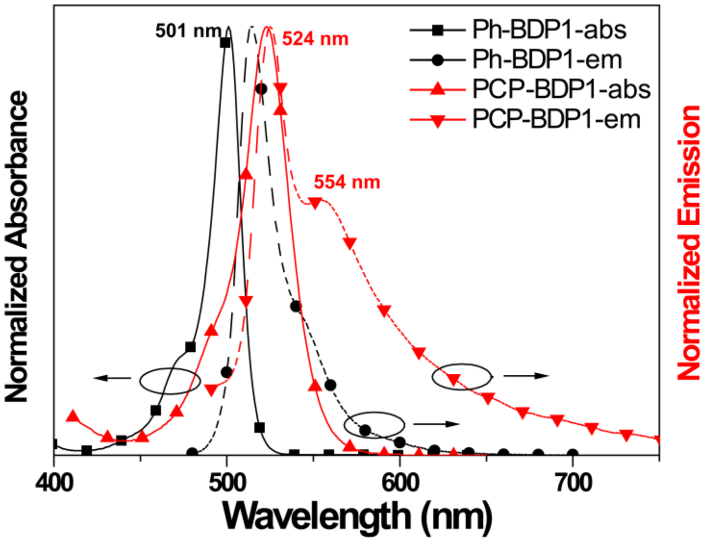

Figure S4. Normalized spectra of Ph-BDP1 and PCP-BDP1 in DCM (1.0×10^{-5} mol L^{-1}).

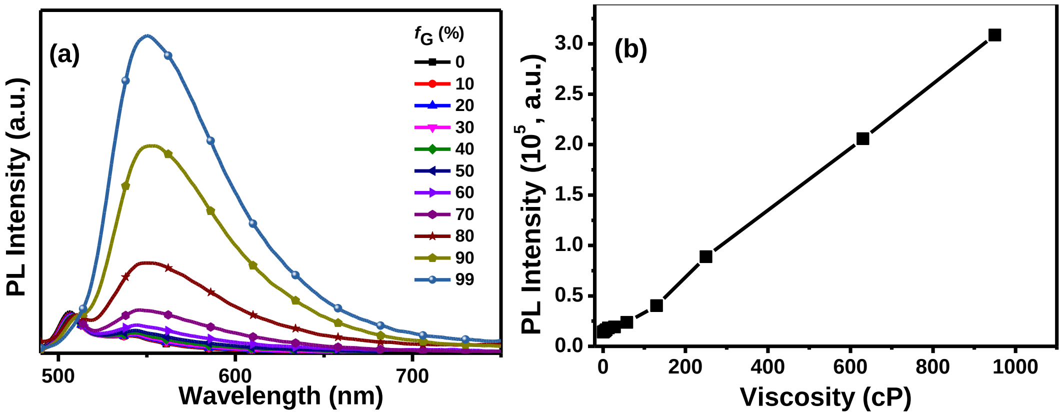

Supplementary Figure 5. (a) Fluorescence spectra of PCP-BDP1 in MeOH/Glycerol binary solvents (10 μM) with varied volumetric fractions of Glycerol (f_G). λ_{ex} = 470 nm. (b) Fluorescence intensity changes at 550 nm *vs* viscosity for PCP-BDP1.

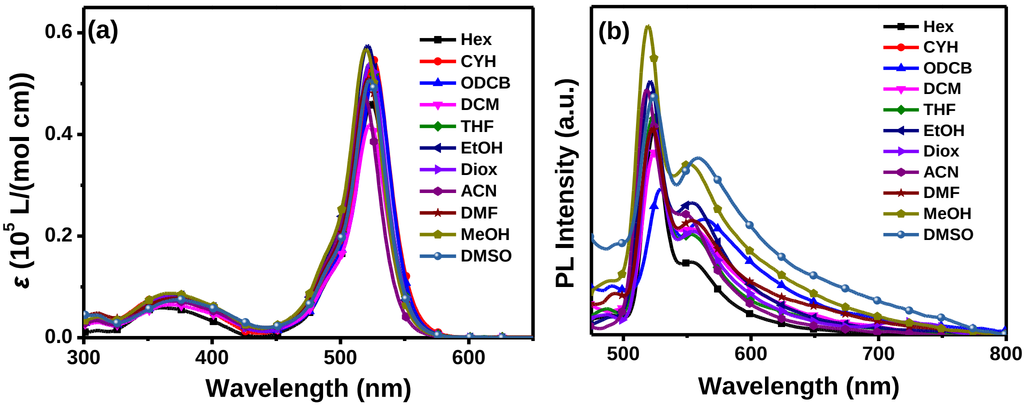

Supplementary Figure 6. Absorption (a) and emission (b) spectra of PCP-BDP1 (10 μM) in various solvents including hexane (Hex), cyclohexane (CYH), 1,2-dichlorobenzene (ODCB), dichloromethane (DCM), tetrahydrofuran (THF), ethanol (EtOH), 1,4-dioxane (Diox), acetonitrile (ACN), *N*,*N*-dimethylformamide (DMF), methanol (MeOH), dimethyl sulfoxide (DMSO), respectively.

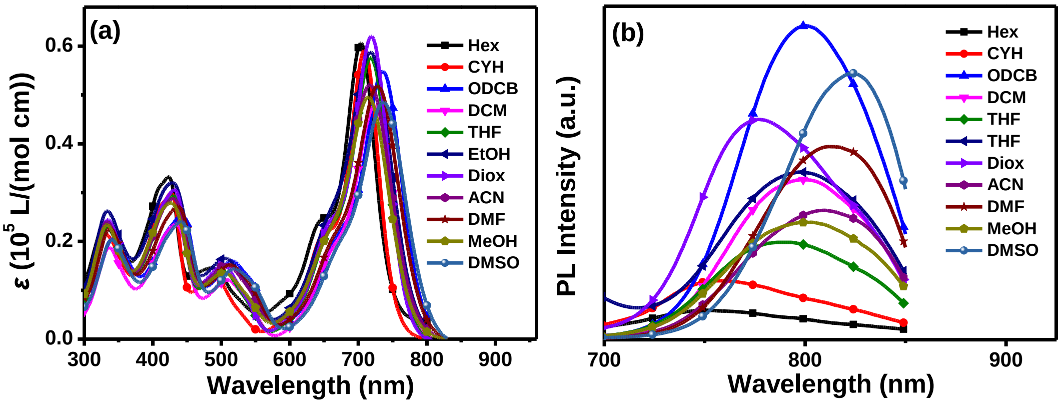

Supplementary Figure 7. Absorption (a) and emission (b) spectra of PCP-BDP2 (10 μM) in various solvents including hexane (Hex), cyclohexane (CYH), 1,2-dichlorobenzene (ODCB), dichloromethane (DCM), tetrahydrofuran (THF), ethanol (EtOH), 1,4-dioxane (Diox), acetonitrile (ACN), *N*,*N*-dimethylformamide (DMF), methanol (MeOH), dimethyl sulfoxide (DMSO), respectively.

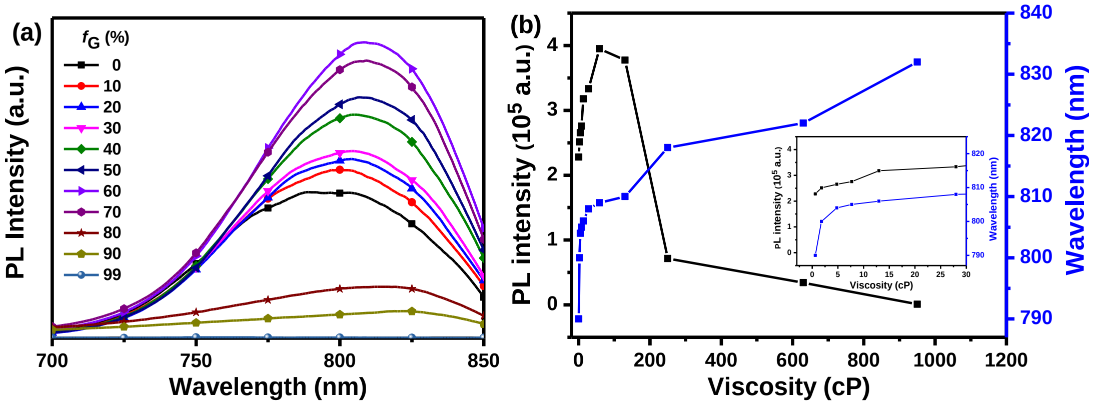

Supplementary Figure 8. (a) Fluorescence spectra of PCP-BDP2 in MeOH/Glycerol binary solvents (10 μM) with varied volumetric fractions of Glycerol (f_G); (b) Emission intensity at 790 nm and wavelength changes of PCP-BDP2 with varied viscosity.

Supplementary Table 3. Calculated excited wavelength (λ) and oscillator strengths (f) and related wave functions

Compound	Electronic transition [a] [b]	Energy [eV]	λ [nm]	f[c]	Orbitals (coefficient)[d]
**Ph-BDP1**	$S_0 \rightarrow S_1$	2.9155	425.26	0.5926	H>L (97%), H-1>L (3%), H<L (2%)
	$S_1 \rightarrow S_0$	2.6772	463.12	0.7293	H>L (99%)
**PCP-BPD1**	$S_0 \rightarrow S_1$	2.6953	460.01	0.2732	H>L (72%), H-1>L (24%), H-3>L (3%)
	$S_0 \rightarrow S_2$	2.8680	432.31	0.2146	H>L (26%), H-1>L (69%), H-3>L (4%)
	$S_1 \rightarrow S_0$	2.1258	583.24	0.0900	H>L (77%), H-1>L (20%)
	$S_2 \rightarrow S_0$	2.5344	489.20	0.4344	H>L (19%), H-1>L (76%), H-2>L (4%)
**Ph-BDP2**	$S_0 \rightarrow S_1$	1.7855	694.38	1.0902	H>L (99%)
	$S_0 \rightarrow S_2$	2.3663	523.97	0.5364	H-1>L (94%), H>L+1 (5%)
	$S_1 \rightarrow S_0$	1.5373	806.53	1.2319	H>L (99%)
**PCP-BPD2**	$S_0 \rightarrow S_1$	1.7083	725.80	0.9949	H>L (99%)
	$S_0 \rightarrow S_2$	2.2836	542.94	0.6569	H-1>L (95%), H>L+1 (4%)
	$S_1 \rightarrow S_0$	1.4361	863.36	1.0867	H>L (99%)

[a] Only selected excited states were considered. [b] DCM was employed as the solvent for the DFT calculations. [c] Oscillator strength. [d] MOs involved in the transitions. H = HOMO; L = LUMO. Coefficient of the wavefunction for each excitation.

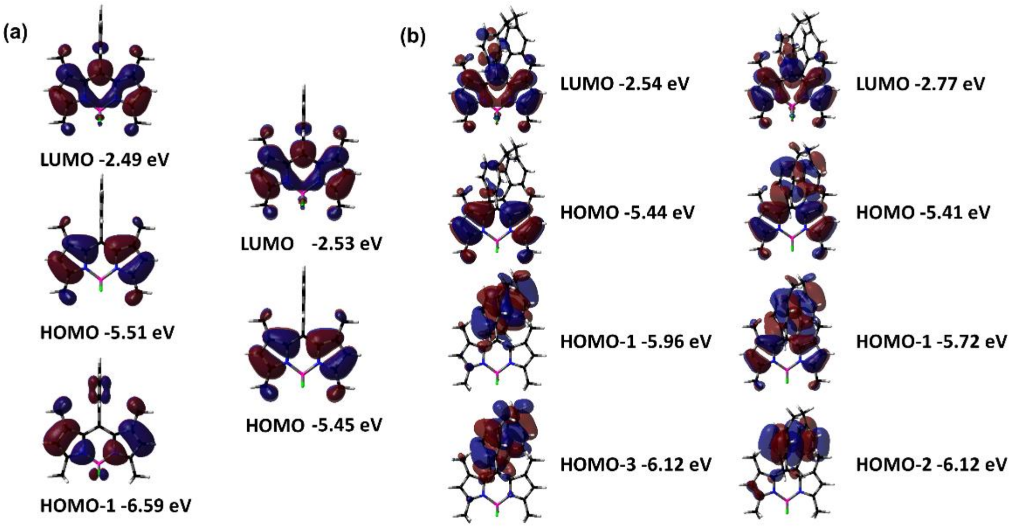

Supplementary Figure 9. Calculated frontier molecular orbitals for Ph-BDP1 and PCP-BDP1 and their orbital energies in the optimized ground (left) and excited state (right).

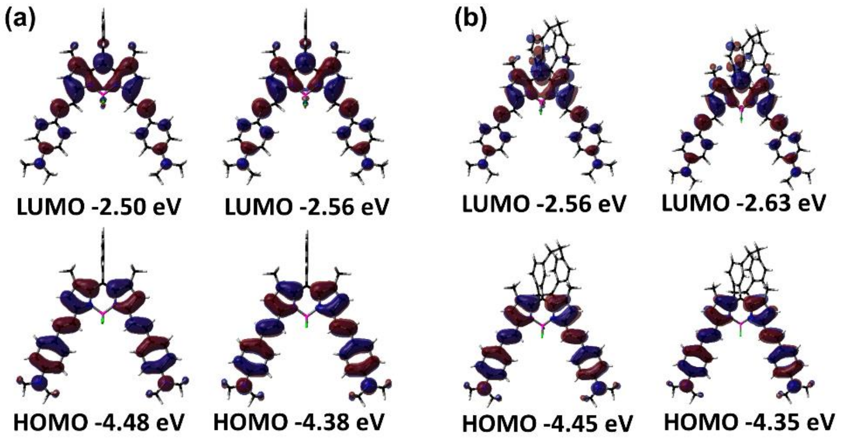

Supplementary Figure 10. Calculated frontier molecular orbitals for Ph-BDP2 and PCP-BDP2 and their orbital energies in the optimized ground (left) and excited state (right).

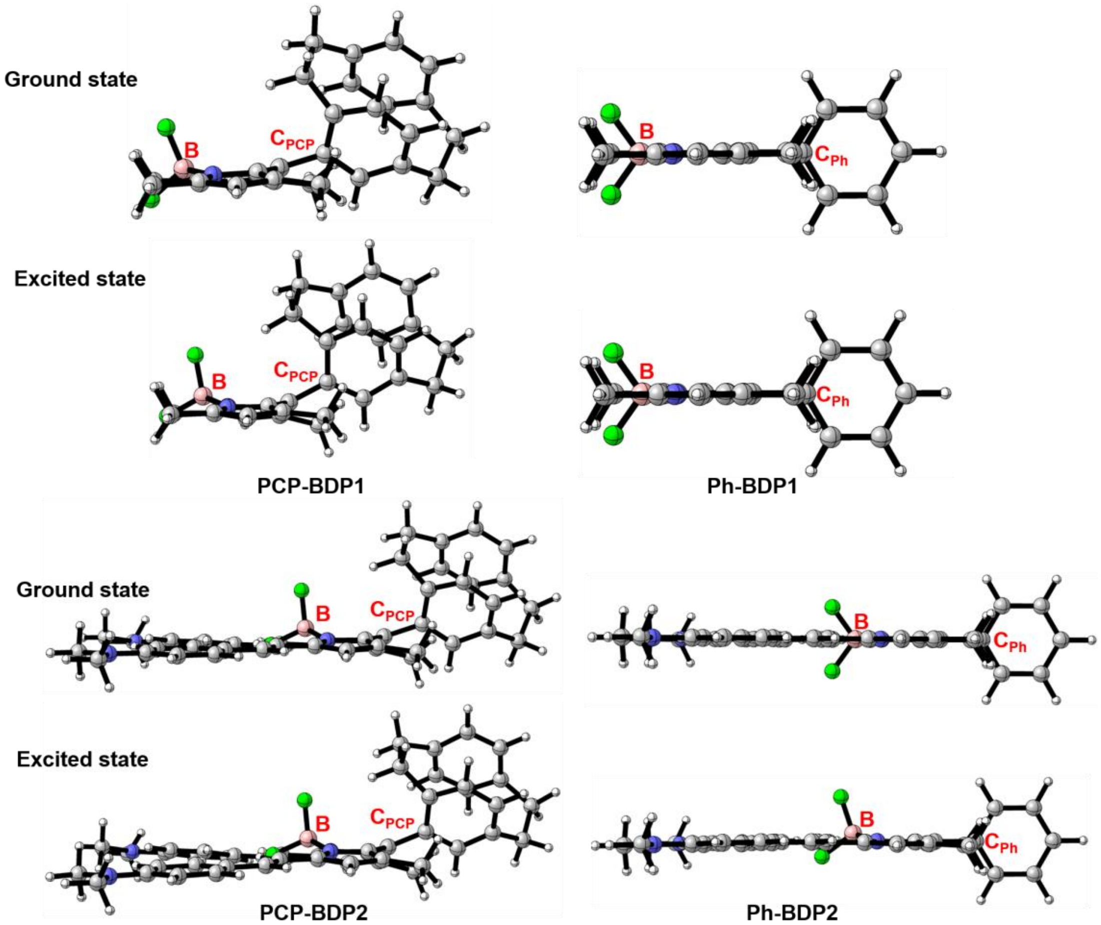

Supplementary Figure 11. Optimazed geometries of Ph-BDP1, Ph-BDP2, PCP-BDP1 and PCP-BDP2 in the ground and excited states.

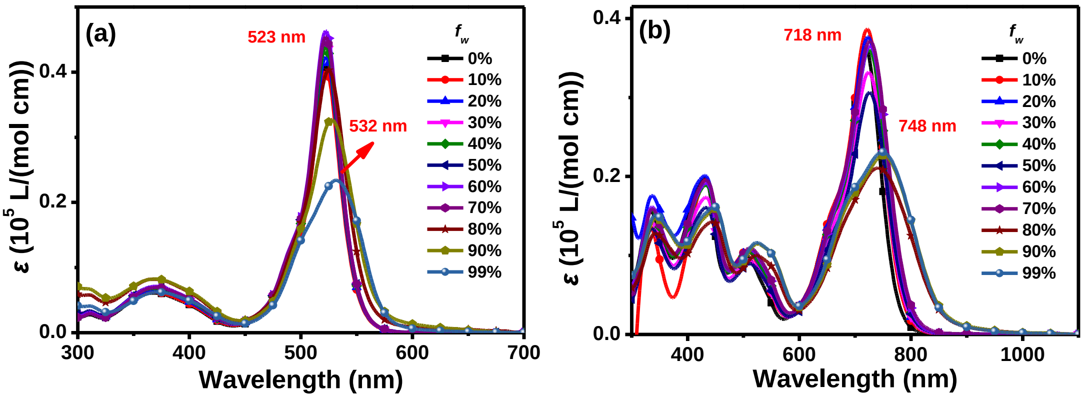

Supplementary Figure 12. Absorption spectra of PCP-BDP1 (a) (10 μM) and PCP-BDP2 (b) (10 μM) in THF-water binary solvents varied f_w.

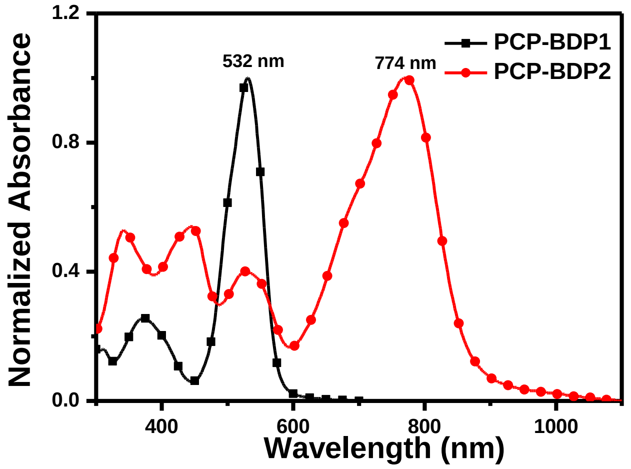

Supplementary Figure 13. Normalized absorption spectra of PCP-BDP1 and PCP-BDP2 thin films. Spin-coating technique was used to prepare the thin films of PCP-BDP1 (0.1 mM in DCM) and PCP-BDP2 (0.1 mM in DCM) in quartz plate.

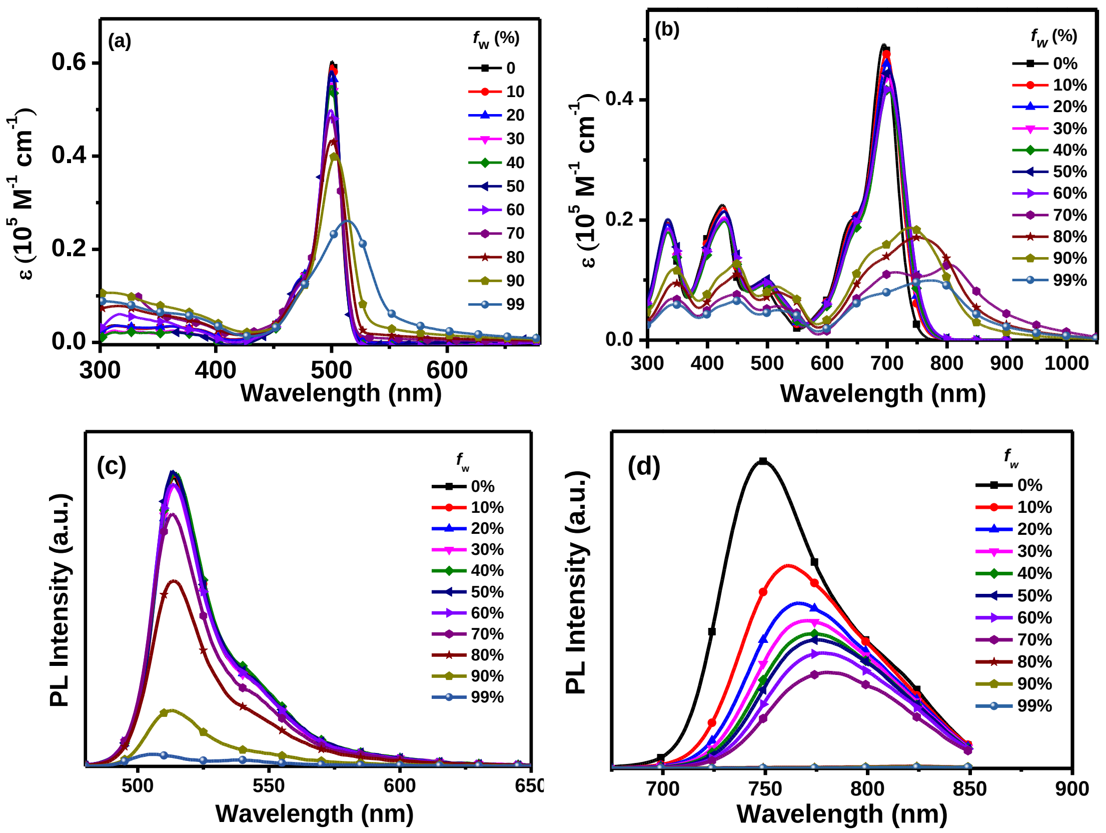

Supplementary Figure 14. Absorption and emission spectra of Ph-BDP1 (a, c) and Ph-BDP2 (b, d) (10 μM) in THF-water mixtures with varied volumetric fractions of water (f_w).

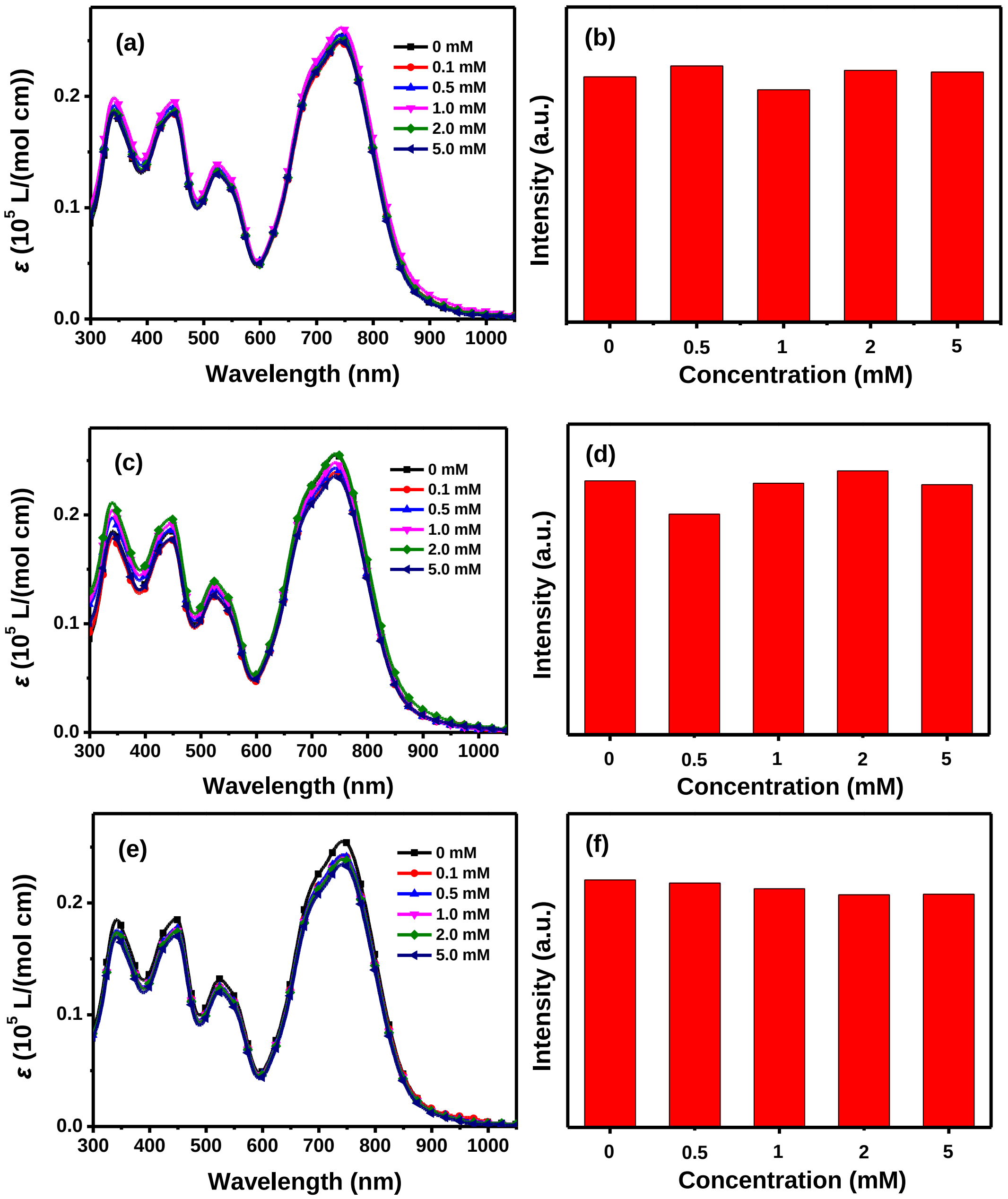

Supplementary Figure 15. Absorption spectra and emission intensity of PCP-BDP2 NPs against cysteine (Cys) (a-b), glutathione (GHS) (c-d), and hydrogen peroxide (H_2O_2) (e-f). PCP-BDP2 NPs were incubated with different medium at room temperature for 6 h.

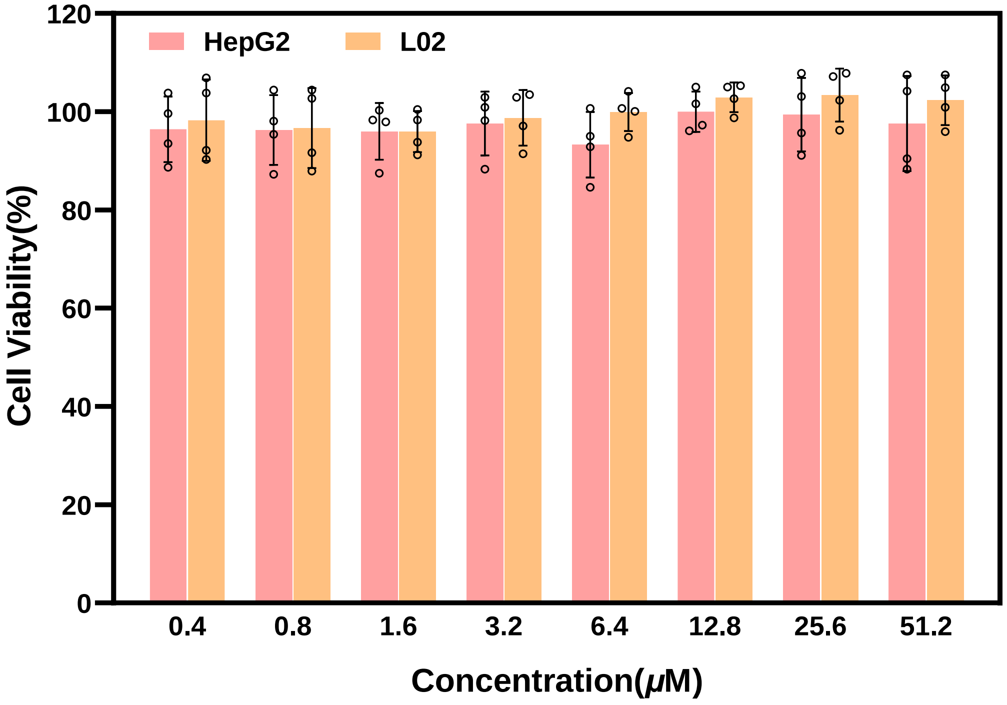

Supplementary Figure 16. Cell viability of L02 and HepG2 cells incubated with PCP-BDP2 at various concentrations. Bars represent mean ± s.d. derived from n = 4 independent experiments.

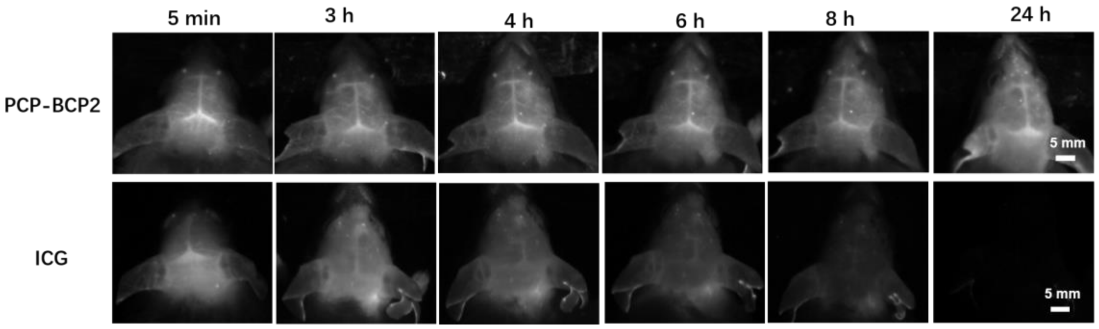

Supplementary Figure 17. The fluorescent imaging of brain blood vessels at other time points of PCP-BDP2 NPs and ICG.

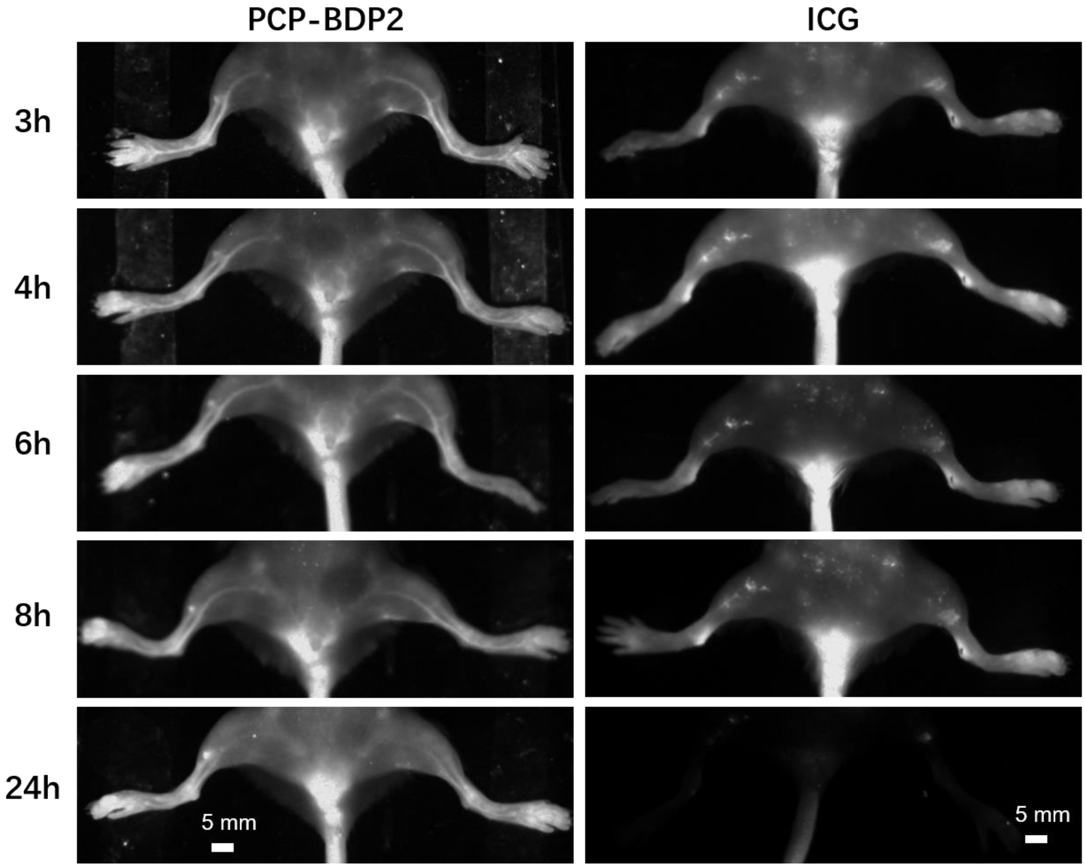

Supplementary Figure 18. The fluorescent imaging of hindlimb vessels at other time points of PCP-BDP2 NPs and ICG.

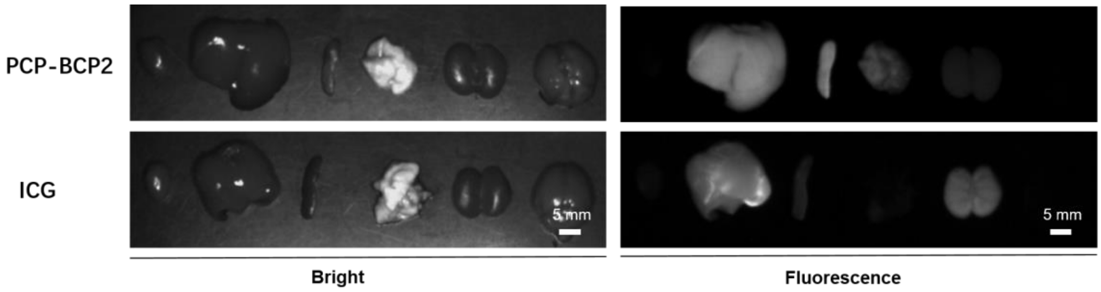

Supplementary Figure 19. In vivo biodistribution of PCP-BDP2 NPs and ICG in mice.

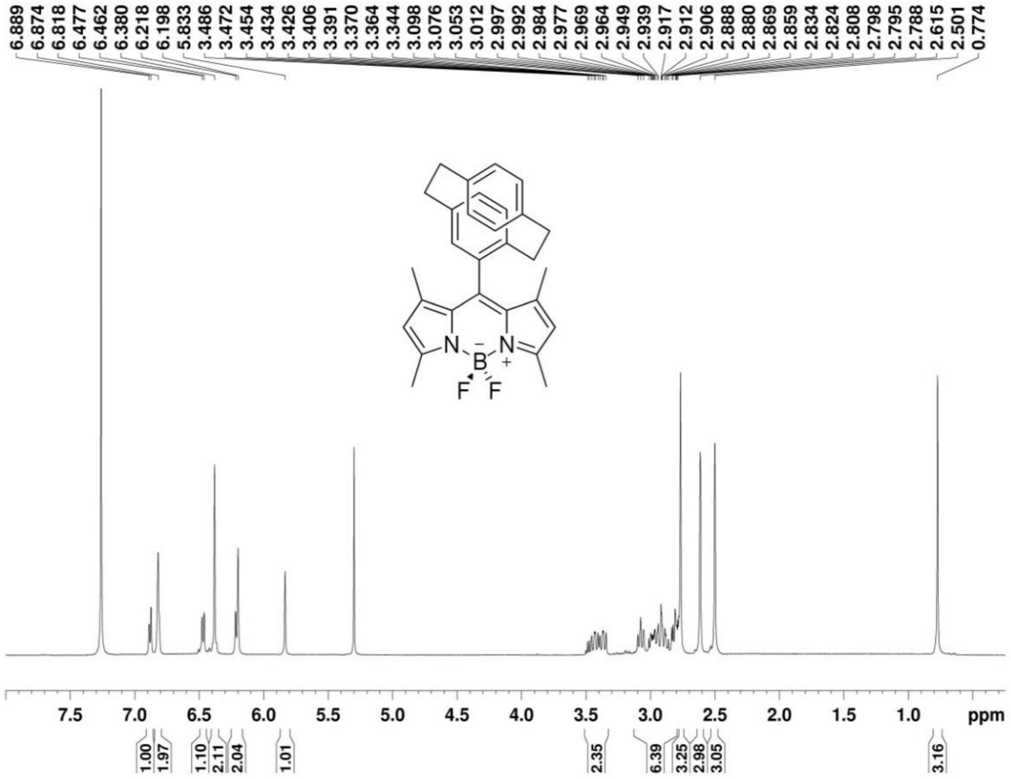

Supplementary Figure 20. ^{1}H NMR spectrum of PCP-BDP1 in $CDCl_3$.

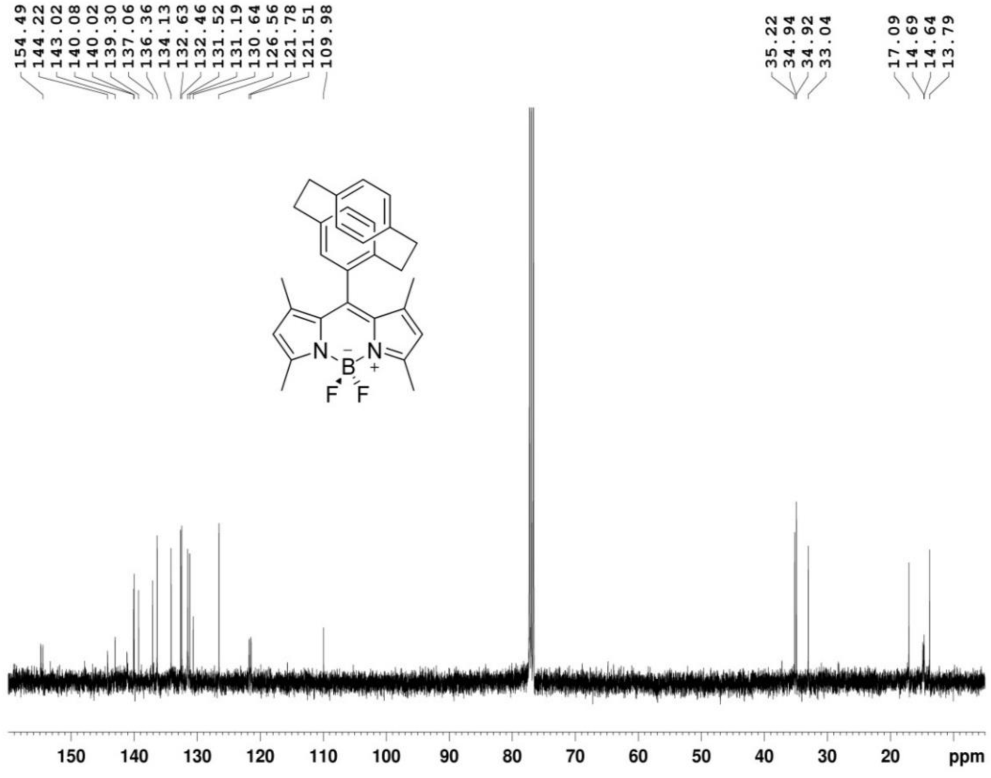

Supplementary Figure 21. ^{13}C NMR spectrum of PCP-BDP1 in $CDCl_3$.

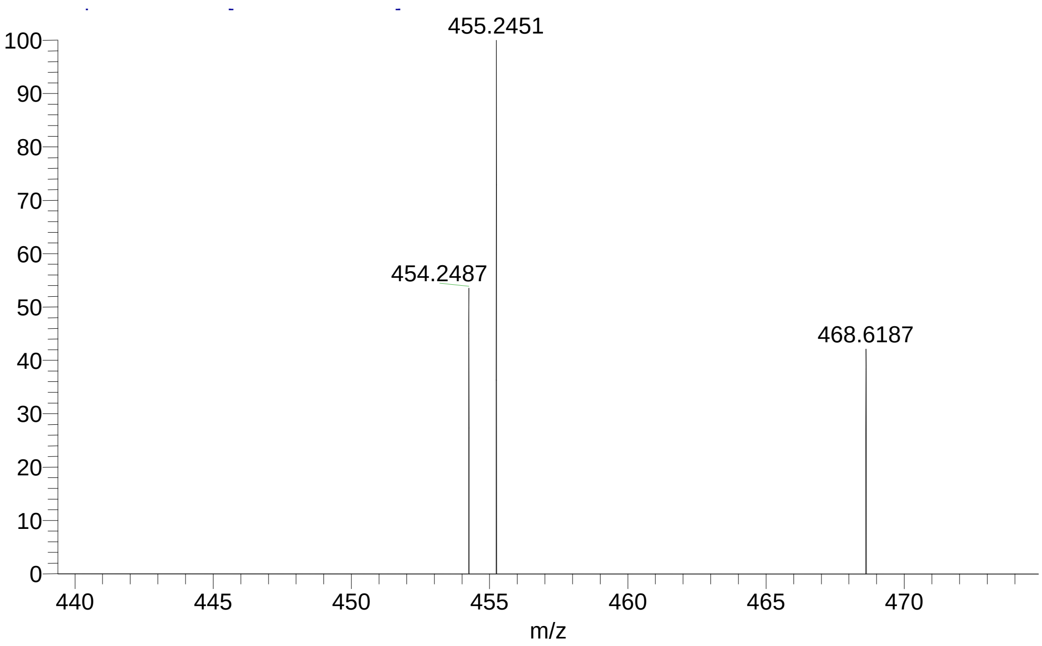

Supplementary Figure 22. HRMS spectrum of PCP-BDP1.

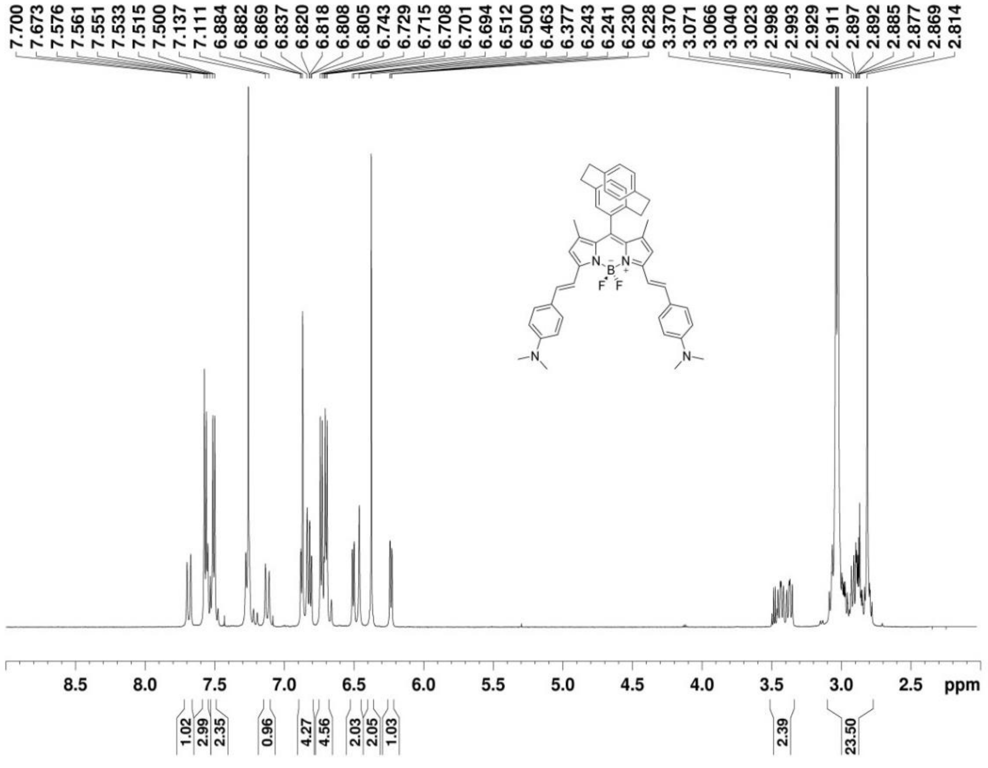

Supplementary Figure 23. ^{1}H NMR spectrum of PCP-BDP2 in $CDCl_3$.

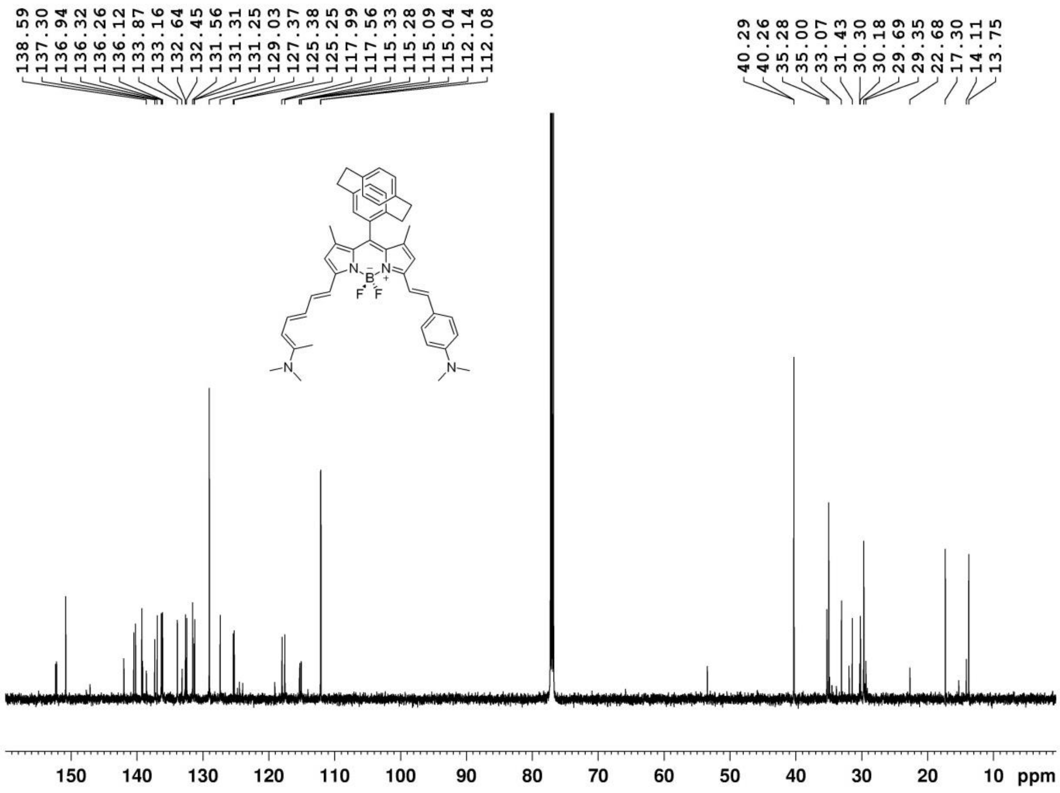

Supplementary Figure 24. ^{13}C NMR spectrum of PCP-BDP2 in $CDCl_3$.

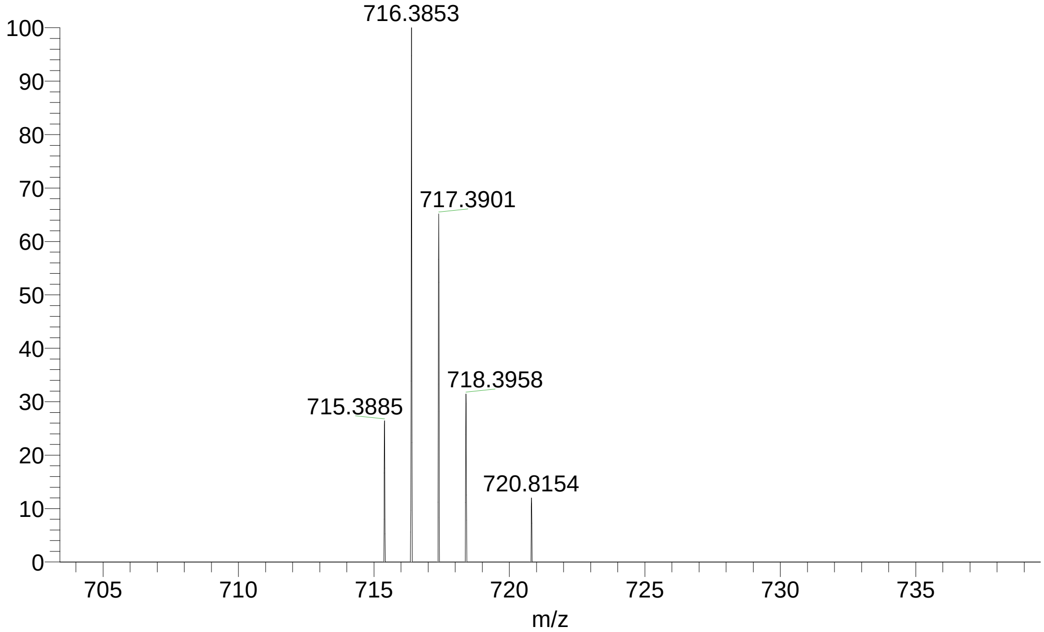

Supplementary Figure 25. HRMS spectrum of PCP-BDP2

7. Coordinates of optimized structures

Coordinates of PCP-BDP1 in the ground state.

F	-3.718600	0.441100	1.734300
F	-4.834500	0.561200	-0.261900
N	-3.010300	-1.002100	-0.084500
N	-2.583300	1.425000	-0.174300
B	-3.588100	0.364000	0.340600
C	-3.673600	-2.179000	-0.125200
C	-2.784500	-3.185600	-0.535900
H	-3.049900	-4.222400	-0.699900
C	-1.526100	-2.618900	-0.727900
C	-1.668900	-1.221800	-0.420300
C	-0.752200	-0.144300	-0.520600
C	0.694100	-0.541100	-0.515800
C	1.179400	-1.270600	0.609500
C	2.340500	-2.023000	0.421700
H	2.667600	-2.687800	1.217300
C	3.182900	-1.807600	-0.665200
H	4.142300	-2.317400	-0.704400
C	2.902600	-0.780500	-1.569100
C	1.585200	-0.282000	-1.571100
H	1.265300	0.358100	-2.387500
C	0.677400	-1.100800	2.031900
H	0.759000	-2.060100	2.553700
H	-0.376600	-0.813600	2.059100
C	1.506400	-0.013100	2.886700
H	0.834900	0.826800	3.093300
H	1.751400	-0.471700	3.851100
C	2.755700	0.498000	2.197200
C	3.961700	-0.216700	2.197800
H	4.121400	-1.005400	2.930500
C	4.870100	-0.076200	1.142600
H	5.709500	-0.765500	1.075100
C	4.604300	0.796600	0.078200
C	3.592200	1.745000	0.282900
H	3.442500	2.529800	-0.454100
C	2.671200	1.588400	1.316800
H	1.804400	2.243900	1.363500
C	5.138300	0.537700	-1.319200
H	5.987900	-0.150800	-1.258000
H	5.507700	1.455800	-1.791900
C	4.041400	-0.092900	-2.303900
H	3.630600	0.706000	-2.930300
H	4.567400	-0.782000	-2.976200

C	-1.239200	1.187400	-0.522200
C	-0.659600	2.462300	-0.841200
C	-1.658600	3.411100	-0.641000
H	-1.566800	4.477300	-0.805500
C	-2.823400	2.753700	-0.218000
C	-5.119900	-2.321900	0.224800
H	-5.325000	-1.917400	1.222400
H	-5.405600	-3.376700	0.210100
H	-5.754600	-1.772800	-0.477800
C	-0.352300	-3.383100	-1.267000
H	0.322100	-3.716900	-0.471300
H	0.248300	-2.785600	-1.957900
H	-0.708700	-4.272100	-1.797400
C	-4.134600	3.370400	0.150700
H	-4.924900	3.067800	-0.543500
H	-4.050500	4.459900	0.137300
H	-4.450200	3.054400	1.150900
C	0.670500	2.809000	-1.434000
H	0.665200	2.658700	-2.522100
H	1.481300	2.209800	-1.030100
H	0.891600	3.866700	-1.256700

Coordinates of PCP-BDP1 in the excited state.

F	-3.097600	0.395700	1.861800
F	-4.715600	0.624600	0.250300
N	-2.966900	-1.008900	-0.111800
N	-2.480300	1.412400	-0.254700
B	-3.356100	0.364500	0.467900
C	-3.668700	-2.186200	-0.033300
C	-2.839600	-3.221700	-0.460100
H	-3.131400	-4.262400	-0.542400
C	-1.582300	-2.685200	-0.800700
C	-1.677900	-1.284800	-0.567300
C	-0.741500	-0.221800	-0.769100
C	0.694300	-0.584200	-0.578700
C	1.077500	-1.060700	0.739200
C	2.211800	-1.900500	0.825700
H	2.408900	-2.433400	1.750900
C	3.162500	-1.887300	-0.173500
H	4.096100	-2.426600	-0.048100
C	2.992900	-1.025900	-1.291400
C	1.682100	-0.555100	-1.554100
H	1.468400	-0.102100	-2.516700
C	0.404700	-0.631700	2.031700

H	0.333800	-1.505800	2.688500
H	-0.610700	-0.278400	1.853500
C	1.226000	0.502500	2.792400
H	0.593200	1.392400	2.860200
H	1.427900	0.167900	3.814200
C	2.522600	0.845000	2.082700
C	3.735700	0.190800	2.370400
H	3.833900	-0.380600	3.289700
C	4.734900	0.114700	1.404000
H	5.599700	-0.520900	1.576100
C	4.549900	0.708200	0.137500
C	3.508800	1.647900	0.013500
H	3.424500	2.231700	-0.898300
C	2.500200	1.705000	0.963600
H	1.625000	2.323300	0.784800
C	5.246300	0.180300	-1.107800
H	6.028300	-0.525400	-0.812400
H	5.734200	0.981100	-1.674800
C	4.209300	-0.543200	-2.059700
H	3.882700	0.148000	-2.841300
H	4.712300	-1.379600	-2.561200
C	-1.209800	1.132400	-0.770200
C	-0.668900	2.350200	-1.272200
C	-1.633600	3.343500	-1.015600
H	-1.557800	4.389300	-1.290400
C	-2.729900	2.753400	-0.389900
C	-5.083300	-2.269300	0.446300
H	-5.190500	-1.895400	1.472500
H	-5.417800	-3.311000	0.428800
H	-5.762500	-1.675200	-0.175000
C	-0.444200	-3.463600	-1.399100
H	0.409900	-3.576400	-0.718500
H	-0.061000	-2.996200	-2.314200
H	-0.779700	-4.472800	-1.662200
C	-3.986600	3.414200	0.081400
H	-4.872200	3.028000	-0.434700
H	-3.923300	4.492200	-0.095600
H	-4.155400	3.253800	1.153700
C	0.554200	2.567000	-2.114700
H	0.453100	2.101200	-3.106100
H	1.465500	2.162000	-1.671500
H	0.712400	3.638500	-2.278400

Coordinates of Ph-BDP1 in the ground state.

C	-1.90258400	2.53042100	0.00002300
C	0.37033800	2.58186600	-0.00025300
C	-0.77447700	3.37292400	-0.00025900
H	-0.80594700	4.45505000	-0.00038200
C	1.77186100	3.11762000	-0.00055500
H	1.74783900	4.21156000	-0.00007400
H	2.33748200	2.79033700	-0.87927400
H	2.33826500	2.78954000	0.87734500
C	-0.08910400	-1.22270000	-0.00002000
C	0.37033800	-2.58186500	0.00003900
C	-0.77447800	-3.37292300	-0.00005500
H	-0.80594800	-4.45504900	-0.00007100
C	-1.90258400	-2.53042100	-0.00023400
C	1.77186000	-3.11762000	0.00028800
H	1.74783800	-4.21155900	-0.00018500
H	2.33751800	-2.79032700	0.87897900
H	2.33822800	-2.78954900	-0.87764000
B	-2.41795700	0.00000000	0.00016900
F	-3.22593700	-0.00026100	1.14442200
F	-3.22647200	0.00026100	-1.14369300
N	-1.49127500	1.24436400	0.00021900
N	-1.49127500	-1.24436400	-0.00030400
C	2.80630200	0.00033400	1.20967500
H	2.26227900	0.00060900	2.15019700
C	2.80637400	-0.00033500	-1.20957600
H	2.26240700	-0.00060900	-2.15013100
C	2.09822900	0.00000000	0.00002800
C	4.20264200	0.00033900	1.20835800
H	4.74131200	0.00059900	2.15187000
C	4.90339300	-0.00000100	0.00011200
H	5.98975800	-0.00000100	0.00014400
C	4.20271400	-0.00034000	-1.20817600
H	4.74144100	-0.00060000	-2.15165500
C	0.60279500	0.00000000	-0.00000400
C	-0.08910400	1.22270000	-0.00002900
C	-3.34417200	2.92333000	0.00020800
H	-3.85657500	2.51937400	-0.87955700
H	-3.85658700	2.51839200	0.87950700
H	-3.44192300	4.01143500	0.00079100
C	-3.34417200	-2.92332900	-0.00044000
H	-3.85665000	-2.51910700	0.87916000
H	-3.85651400	-2.51865700	-0.87990400

Coordinates of Ph-BDP1 in the excited state.

C	-1.92079600	2.52871600	0.00022100
C	0.36179900	2.60124100	-0.00033200
C	-0.78975100	3.37931500	-0.00014100
H	-0.83236400	4.46172000	-0.00023700
C	1.76367300	3.12861200	-0.00084400
H	1.74452000	4.22305900	-0.00039100
H	2.33239000	2.80065800	-0.87921900
H	2.33337700	2.79990700	0.87658500
C	-0.09279700	-1.22575400	-0.00004500
C	0.36180000	-2.60124100	0.00005200
C	-0.78974900	-3.37931500	-0.00018300
H	-0.83236200	-4.46172000	-0.00021700
C	-1.92079500	-2.52871600	-0.00039900
C	1.76367400	-3.12860900	0.00044600
H	1.74452300	-4.22305700	-0.00000300
H	2.33246500	-2.80064900	0.87876900
H	2.33330400	-2.79991000	-0.87703500
B	-2.42167700	-0.00000100	0.00015800
F	-3.23846000	-0.00031500	1.14255100
F	-3.23891600	0.00031300	-1.14189900
N	-1.49671700	1.23590300	0.00030600
N	-1.49671600	-1.23590300	-0.00036600
C	2.83578600	0.00006000	1.20798400
H	2.29247100	0.00011600	2.14909100
C	2.83590900	-0.00006000	-1.20784600
H	2.29269100	-0.00011600	-2.14900900
C	2.12118400	0.00000000	0.00003300
C	4.23232500	0.00005500	1.20808700
H	4.77109600	0.00009800	2.15175100
C	4.93363500	0.00000000	0.00017600
H	6.02013000	0.00000000	0.00023100
C	4.23244700	-0.00005500	-1.20780700
H	4.77131500	-0.00009800	-2.15141600
C	0.62881500	0.00000000	-0.00003000
C	-0.09279700	1.22575500	-0.00005800
C	-3.35825100	2.90585100	0.00055400
H	-3.87257900	2.49055500	-0.87655800
H	-3.87237800	2.48961300	0.87732300
H	-3.47101100	3.99243300	0.00111300
C	-3.35824900	-2.90585200	-0.00067300
H	-3.87258200	-2.49032100	0.87632300
H	-3.87237300	-2.48985000	-0.87755800
H	-3.47100900	-3.99243500	-0.00095200

Coordinates of PCP-BDP2 in the ground state.

C	-7.37190000	6.20360000	0.70130000
H	-8.19660000	6.90840000	0.81080000
H	-7.24660000	5.67220000	1.65520000
H	-7.65560000	5.46870000	-0.06300000
C	-6.19410000	8.38730000	0.29260000
H	-7.21450000	8.73160000	0.46340000
H	-5.55030000	8.82460000	1.06900000
H	-5.86520000	8.77530000	-0.68000000
C	-4.98930000	6.25840000	0.11270000
C	-4.92090000	4.84090000	0.19470000
H	-5.80830000	4.26650000	0.43190000
C	-3.73310000	4.16520000	-0.02240000
H	-3.73780000	3.08160000	0.05360000
C	-2.53240000	4.84230000	-0.33230000
C	-2.60670000	6.24930000	-0.40830000
H	-1.70480000	6.81160000	-0.64090000
C	-3.78660000	6.94450000	-0.19420000
H	-3.77540000	8.02550000	-0.26350000
C	-1.26220000	4.18140000	-0.56370000
H	-0.43700000	4.85720000	-0.78600000
C	-1.00940000	2.84460000	-0.52560000
H	-1.80610000	2.14780000	-0.29930000
C	0.28840000	2.27340000	-0.77410000
C	1.94690000	0.74480000	-0.86490000
C	2.50180000	2.01400000	-1.25320000
C	1.46880000	2.93310000	-1.19240000
H	1.54430000	3.97760000	-1.46420000
C	3.85040000	2.35000000	-1.81330000
H	3.98950000	3.43610000	-1.81600000
H	3.94310000	2.00830000	-2.85290000
H	4.66420000	1.89790000	-1.25180000
C	2.48630000	-0.56280000	-0.74950000
C	1.60290000	-1.66980000	-0.71040000
C	1.83630000	-3.06680000	-0.97040000
C	0.59910000	-3.68430000	-0.90320000
H	0.40810000	-4.73170000	-1.09640000
C	-0.38580000	-2.71240000	-0.58990000
C	3.10200000	-3.77090000	-1.36480000
H	2.86060000	-4.73840000	-1.81660000
H	3.75600000	-3.95200000	-0.50560000
H	3.68600000	-3.19090000	-2.08550000
C	-1.79570000	-2.89960000	-0.37640000
H	-2.37790000	-2.00000000	-0.22120000

C	-2.40400000	-4.11730000	-0.36640000
H	-1.78890000	-5.00310000	-0.52200000
C	-3.81410000	-4.39130000	-0.16810000
C	-4.27500000	-5.72450000	-0.20460000
H	-3.55850000	-6.52400000	-0.38140000
C	-5.60910000	-6.05520000	-0.02640000
H	-5.89850000	-7.09830000	-0.06910000
C	-6.58240000	-5.05050000	0.20790000
C	-6.12530000	-3.70480000	0.24500000
H	-6.82650000	-2.89710000	0.41730000
C	-4.78910000	-3.39520000	0.06380000
H	-4.49450000	-2.35030000	0.10330000
C	-8.89050000	-4.30350000	0.58550000
H	-9.87480000	-4.75130000	0.72550000
H	-8.94190000	-3.62850000	-0.28010000
H	-8.66360000	-3.70070000	1.47430000
C	3.92900000	-0.89260000	-0.50310000
C	4.97800000	-0.65870000	-1.40600000
H	4.77810000	-0.10140000	-2.31560000
C	6.29830000	-1.06930000	-1.13540000
C	6.46880000	-2.00440000	-0.11270000
H	7.44590000	-2.44930000	0.05990000
C	5.45600000	-2.19950000	0.82370000
H	5.67080000	-2.78130000	1.71680000
C	4.24330000	-1.51080000	0.74370000
C	-8.35390000	-6.74430000	0.30060000
H	-9.42840000	-6.78670000	0.48090000
H	-7.85950000	-7.37730000	1.04910000
H	-8.15650000	-7.17300000	-0.69190000
C	7.50920000	-0.36800000	-1.72640000
H	7.17690000	0.32440000	-2.50730000
H	8.19520000	-1.07750000	-2.20550000
C	8.35970000	0.45250000	-0.64080000
H	9.23290000	-0.14600000	-0.35950000
H	8.73920000	1.35260000	-1.13930000
C	7.56270000	0.79100000	0.60560000
C	6.47520000	1.67260000	0.53200000
H	6.41740000	2.37680000	-0.29400000
C	5.38820000	1.53070000	1.39240000
H	4.48760000	2.11430000	1.21450000
C	5.37470000	0.53040000	2.37720000
C	6.60020000	-0.08730000	2.66350000
H	6.66900000	-0.79540000	3.48700000
C	7.67810000	0.03340000	1.77970000

H	8.55540000	-0.59300000	1.92990000
C	4.05250000	-0.01200000	2.88170000
H	3.29800000	0.78060000	2.84210000
H	4.12930000	-0.32950000	3.92770000
C	3.48890000	-1.26410000	2.03880000
H	3.57570000	-2.15390000	2.67140000
H	2.42250000	-1.09430000	1.87090000
B	-0.32240000	-0.14960000	0.01090000
F	-0.23650000	-0.10300000	1.41120000
F	-1.65320000	0.01940000	-0.39400000
N	-6.17020000	6.93210000	0.32040000
N	-7.90870000	-5.36160000	0.39310000
N	0.58090000	0.94640000	-0.61870000
N	0.23440000	-1.49620000	-0.51130000

Coordinates of PCP-BDP2 in the excited state.

C	-7.29070000	6.07110000	0.83480000
H	-8.11570000	6.76570000	0.99180000
H	-7.10830000	5.53770000	1.77680000
H	-7.60040000	5.33730000	0.07940000
C	-6.19900000	8.26540000	0.26620000
H	-7.21320000	8.58820000	0.50090000
H	-5.50900000	8.77780000	0.94950000
H	-5.96730000	8.58450000	-0.75820000
C	-4.93580000	6.16600000	0.14940000
C	-4.82620000	4.75180000	0.27800000
H	-5.68510000	4.16640000	0.58320000
C	-3.63830000	4.09830000	0.01950000
H	-3.61150000	3.01880000	0.13330000
C	-2.46780000	4.79330000	-0.38420000
C	-2.58830000	6.20090000	-0.50970000
H	-1.71650000	6.77470000	-0.81600000
C	-3.76950000	6.87120000	-0.25610000
H	-3.79420000	7.94820000	-0.36920000
C	-1.20540000	4.16210000	-0.65830000
H	-0.40650000	4.84070000	-0.95400000
C	-0.92310000	2.81900000	-0.57240000
H	-1.70200000	2.13130000	-0.26810000
C	0.34680000	2.24320000	-0.85640000
C	1.99250000	0.69290000	-0.96920000
C	2.53130000	1.94320000	-1.45030000
C	1.50940000	2.87300000	-1.36220000
H	1.57610000	3.90640000	-1.67790000
C	3.84910000	2.23630000	-2.10220000
H	3.92060000	3.30720000	-2.31940000

H	3.95460000	1.70280000	-3.05660000
H	4.70180000	1.95830000	-1.48330000
C	2.54770000	-0.61980000	-0.80910000
C	1.63190000	-1.71390000	-0.76120000
C	1.84440000	-3.11530000	-1.03950000
C	0.60690000	-3.72340000	-0.92870000
H	0.40020000	-4.76910000	-1.11850000
C	-0.36520000	-2.74510000	-0.58820000
C	3.10180000	-3.80270000	-1.48400000
H	2.86060000	-4.78360000	-1.90730000
H	3.80780000	-3.95340000	-0.65990000
H	3.63220000	-3.22260000	-2.24680000
C	-1.75970000	-2.90990000	-0.36550000
H	-2.32260000	-2.00090000	-0.19120000
C	-2.41130000	-4.12070000	-0.36620000
H	-1.82360000	-5.02290000	-0.53100000
C	-3.81880000	-4.34080000	-0.16950000
C	-4.33130000	-5.66260000	-0.19840000
H	-3.64210000	-6.48700000	-0.36720000
C	-5.67300000	-5.94360000	-0.02240000
H	-6.00000000	-6.97570000	-0.05790000
C	-6.61340000	-4.90100000	0.20030000
C	-6.10910000	-3.56970000	0.23310000
H	-6.78330000	-2.73810000	0.39990000
C	-4.76550000	-3.30710000	0.05470000
H	-4.43540000	-2.27320000	0.08840000
C	-8.89230000	-4.07450000	0.58630000
H	-9.89410000	-4.48820000	0.70140000
H	-8.90400000	-3.38010000	-0.26410000
H	-8.65220000	-3.50290000	1.49220000
C	3.96910000	-0.95400000	-0.49720000
C	5.06800000	-0.70140000	-1.33580000
H	4.91230000	-0.13710000	-2.24890000
C	6.37770000	-1.09110000	-0.99860000
C	6.51330000	-2.02590000	0.03050000
H	7.48910000	-2.44960000	0.25780000
C	5.45080000	-2.24750000	0.90560000
H	5.62830000	-2.82650000	1.80940000
C	4.23080000	-1.58010000	0.76020000
C	-8.43760000	-6.53570000	0.32960000
H	-9.51440000	-6.53600000	0.49770000
H	-7.97020000	-7.15570000	1.10550000
H	-8.24280000	-7.00220000	-0.64510000
C	7.59750000	-0.36110000	-1.53360000

H	7.28920000	0.29690000	-2.35330000
H	8.33890000	-1.05590000	-1.94790000
C	8.34460000	0.52230000	-0.42400000
H	9.20980000	-0.04030000	-0.05690000
H	8.73580000	1.41520000	-0.92710000
C	7.44670000	0.87810000	0.74590000
C	6.34120000	1.71940000	0.56200000
H	6.32130000	2.39380000	-0.29050000
C	5.19690000	1.56720000	1.34350000
H	4.29420000	2.11100000	1.07390000
C	5.14050000	0.59760000	2.35710000
C	6.35890000	0.02680000	2.75170000
H	6.38650000	-0.65570000	3.59900000
C	7.49480000	0.15600000	1.94690000
H	8.37640000	-0.43810000	2.18020000
C	3.80280000	0.02200000	2.77760000
H	3.01550000	0.75570000	2.57460000
H	3.77540000	-0.18780000	3.85360000
C	3.41150000	-1.33730000	2.01690000
H	3.58600000	-2.16870000	2.70870000
H	2.33670000	-1.29800000	1.82480000
B	-0.22950000	-0.17150000	0.02200000
F	-0.09060000	-0.11320000	1.42130000
F	-1.58050000	0.01120000	-0.31980000
N	-6.11460000	6.81720000	0.40560000
N	-7.94780000	-5.16420000	0.37550000
N	0.65430000	0.90640000	-0.65670000
N	0.28500000	-1.52390000	-0.51470000

Coordinates of Ph-BDP2 in the ground state.

C	6.88268900	5.23195700	0.00777300
H	7.81061200	5.80423500	-0.00462600
H	6.87972100	4.57131800	-0.86932800
H	6.88215600	4.60262700	0.90827100
C	5.99856400	7.58770500	0.00904900
H	7.07383400	7.76795300	-0.00076200
H	5.56304500	8.08379100	-0.86858800
H	5.58160700	8.06045200	0.90890500
C	4.46765700	5.67130000	-0.00562700
C	4.19920700	4.27509900	-0.00290700
H	5.01688300	3.56439000	-0.00105700
C	2.90230500	3.79334400	-0.00192300
H	2.75541900	2.71710100	0.00014100
C	1.78309700	4.65586400	-0.00258700

C	2.05682700	6.04020200	-0.00344500
H	1.22437200	6.74082900	-0.00285600
C	3.34838700	6.54269700	-0.00437000
H	3.49110200	7.61655700	-0.00398500
C	0.40656200	4.20019500	-0.00171300
H	-0.33392800	4.99946400	-0.00142200
C	-0.03689000	2.91357700	-0.00145200
H	0.67624600	2.09708600	-0.00201600
C	-1.42696100	2.54544500	-0.00088000
C	-3.70725000	2.58454500	-0.00067500
C	-2.57591100	3.38074000	-0.00120000
H	-2.56720800	4.46271900	-0.00180900
C	-5.11255900	3.11279100	-0.00085900
H	-5.09407300	4.20708000	0.00019200
H	-5.67753300	2.78186300	0.87680600
H	-5.67660600	2.78360700	-0.87980000
C	-3.24265800	-1.22240100	0.00041300
C	-3.70715900	-2.58461000	-0.00028600
C	-2.57579400	-3.38076300	-0.00011700
H	-2.56705700	-4.46274100	-0.00057800
C	-1.42686800	-2.54542300	0.00067900
C	-5.11245000	-3.11290000	-0.00112700
H	-5.09392900	-4.20718800	-0.00290500
H	-5.67703300	-2.78140500	-0.87882700
H	-5.67690700	-2.78431600	0.87777800
C	-0.03679200	-2.91352000	0.00118400
H	0.67633800	-2.09702300	0.00187400
C	0.40667600	-4.20013700	0.00073700
H	-0.33381000	-4.99940900	0.00005900
C	1.78320500	-4.65580500	0.00113700
C	2.05692900	-6.04014900	0.00132200
H	1.22446800	-6.74076800	0.00085600
C	3.34848300	-6.54265100	0.00170100
H	3.49121300	-7.61651000	0.00123500
C	4.46775300	-5.67124600	0.00254100
C	4.19931500	-4.27503500	0.00127800
H	5.01701000	-3.56434400	0.00030100
C	2.90241600	-3.79328200	0.00087300
H	2.75552400	-2.71703800	-0.00012400
C	6.88280000	-5.23182800	-0.00537800
H	7.81070200	-5.80422600	0.00131300
H	6.88062000	-4.57801600	0.87697800
H	6.88151500	-4.59550400	-0.90080600
C	5.99866400	-7.58770200	-0.00613700

H	7.07398000	-7.76783800	-0.00039000
H	5.57784000	-8.06545900	-0.90141400
H	5.56698200	-8.07894000	0.87623800
B	-0.92682100	0.00001800	0.00266400
F	-0.10866800	-0.00043100	-1.13622300
F	-0.11432500	0.00049300	1.14562000
N	5.75495200	6.15276400	-0.00990900
N	5.75502300	-6.15269100	0.00480600
N	-1.84708100	1.24739700	-0.00002000
N	-1.84703600	-1.24739400	0.00099000
C	-6.14459300	0.00113100	-1.20860900
H	-5.60040000	0.00207000	-2.14916500
C	-6.14491400	-0.00128000	1.20842500
H	-5.60097100	-0.00220500	2.14912500
C	-5.43393300	-0.00006300	0.00000300
C	-7.54110500	0.00107800	-1.20835000
H	-8.07968500	0.00197500	-2.15208000
C	-8.24243700	-0.00010400	-0.00036900
H	-9.32892500	-0.00011900	-0.00051400
C	-7.54142700	-0.00126900	1.20779700
H	-8.08025700	-0.00218200	2.15138500
C	-3.93794000	-0.00003800	0.00024200
C	-3.24270100	1.22235700	-0.00003400

Coordinates of Ph-BDP2 in the excited state.

C	-6.90136600	-5.08822900	-0.07044500
H	-7.84160200	-5.63929500	-0.07167600
H	-6.87234600	-4.45841700	-0.96918300
H	-6.89067800	-4.43145900	0.80908000
C	-6.07585400	-7.46464800	-0.03084500
H	-7.15496100	-7.61559900	-0.05227800
H	-5.64227000	-7.96836700	-0.90456900
H	-5.68043200	-7.94551500	0.87345500
C	-4.49852100	-5.58559100	-0.03076300
C	-4.19597000	-4.19388700	-0.04908000
H	-4.99685700	-3.46465100	-0.07261000
C	-2.89128800	-3.74384400	-0.03784000
H	-2.71833400	-2.67212300	-0.05230900
C	-1.78598900	-4.63532300	-0.00647100
C	-2.09902100	-6.01898000	0.01432400
H	-1.28388800	-6.73875900	0.03991100
C	-3.39895500	-6.48674100	0.00213300
H	-3.57027900	-7.55621500	0.01870200
C	-0.41110800	-4.21791000	0.00500300
H	0.31620200	-5.02797100	0.03820400

C	0.05528000	-2.92307200	-0.02185800
H	-0.65319400	-2.10346100	-0.05728000
C	1.42703500	-2.55592300	-0.02636800
C	3.71078400	-2.59180400	-0.09118000
C	2.57911400	-3.38504600	-0.08975400
H	2.56909400	-4.46600200	-0.14995800
C	5.11642000	-3.11312300	-0.16778200
H	5.10189800	-4.20793400	-0.18029600
H	5.72646700	-2.79294700	0.68401400
H	5.63754000	-2.77297900	-1.06978100
C	3.24705000	1.22340000	-0.02618600
C	3.71098100	2.59159500	-0.09111900
C	2.57936800	3.38492300	-0.08975000
H	2.56943300	4.46588400	-0.14987100
C	1.42722600	2.55588000	-0.02648800
C	5.11666900	3.11280400	-0.16752900
H	5.10228200	4.20762800	-0.17910000
H	5.63757400	2.77338500	-1.06993100
H	5.72683400	2.79180600	0.68386700
C	0.05549100	2.92311000	-0.02200400
H	-0.65302500	2.10353100	-0.05735500
C	-0.41082900	4.21797300	0.00472500
H	0.31652400	5.02800500	0.03770000
C	-1.78569300	4.63544800	-0.00667200
C	-2.09865900	6.01912800	0.01347800
H	-1.28348700	6.73888700	0.03840500
C	-3.39857600	6.48693900	0.00136500
H	-3.56984300	7.55643300	0.01721500
C	-4.49819100	5.58581800	-0.03074200
C	-4.19570300	4.19409100	-0.04844900
H	-4.99662800	3.46487400	-0.07126000
C	-2.89103800	3.74400100	-0.03730600
H	-2.71813100	2.67226400	-0.05117100
C	-6.90104400	5.08852200	-0.07046000
H	-7.84125100	5.63963100	-0.07306700
H	-6.89123300	4.43242300	0.80959800
H	-6.87118900	4.45802100	-0.96866600
C	-6.07549000	7.46490700	-0.02977100
H	-7.15463600	7.61586000	-0.04913300
H	-5.64350100	7.96896100	-0.90411100
H	-5.67842200	7.94543100	0.87397000
B	0.98556000	0.00001000	0.29615400
F	-0.15031900	0.00003200	-0.54154000
F	0.55359100	0.00008800	1.63358000

N	-5.79370200	-6.03504200	-0.04402600
N	-5.79336100	6.03530600	-0.04386200
N	1.86070900	-1.24028100	0.00865900
N	1.86080200	1.24020700	0.00854800
C	6.17365300	0.00045000	-1.23009200
H	5.63117400	0.00095100	-2.17189900
C	6.17115500	-0.00080100	1.18264300
H	5.62613700	-0.00125900	2.12289900
C	5.45523000	-0.00016000	-0.02453000
C	7.57025900	0.00040300	-1.22982200
H	8.10990700	0.00086800	-2.17324900
C	8.27123700	-0.00024200	-0.02161000
H	9.35790500	-0.00027300	-0.02057900
C	7.56800100	-0.00084000	1.18529900
H	8.10565800	-0.00133200	2.12985700
C	3.96024700	-0.00011100	-0.01992000
C	3.24695900	-1.22357400	-0.02611900

8. References

1. (a) Kollmannsberger, M.; Gareis, T.; Heinl, S.; Breu, J.; Daub, J., Electrogenerated Chemiluminescence and Proton-Dependent Switching of Fluorescence: Functionalized Difluoroboradiaza-s-indacenes. *Angew. Chem. Int. Ed.* **1997**, *36* (12), 1333-1335; (b) Deniz, E.; Isbasar, G. C.; Bozdemir, O. A.; Yildirim, L. T.; Siemiarczuk, A.; Akkaya, E. U., Bidirectional switching of near IR emitting boradiazaindacene fluorophores. *Org. Lett.* **2008**, *10* (16), 3401-3.

2. Friedmann, C. J.; Ay, S.; Brase, S., Improved synthesis of enantiopure 4-hydroxy[2.2]paracyclophane. *J. Org. Chem.* **2010**, *75* (13), 4612-4.

3. Sheldrick, G. M., Crystal structure refinement with SHELXL. *Acta Crystallogr C Struct Chem* **2015**, *71* (Pt 1), 3-8.

4. CYLview20; Legault, C. Y., Université de Sherbrooke, 2020 (http://www.cylview.org)

5. Frisch, M. J., *et al.* Gaussian 09, Revision D.01; Gaussian, Inc.: Wallingford, CT, 2013.
