# Supplementary material for: J-aggregates of meso-[2.2]paracyclophanyl-BODIPY dye for NIR-II imaging
Source: Nat Commun. 2021 Apr 22;12:2376. doi: 10.1038/s41467-021-22686-z (PMC8062432; doi:10.1038/s41467-021-22686-z)
Supplement: Supplementary file 3 — Reporting Summary [file 41467_2021_22686_MOESM3_ESM.pdf]

nature research

Corresponding author(s): Zhipeng Liu, Guo-qiang Zhang, Yuncong Chen

Last updated by author(s): Mar 11, 2021

Reporting Summary

Nature Research wishes to improve the reproducibility of the work that we publish. This form provides structure for consistency and transparency in reporting. For further information on Nature Research policies, see our Editorial Policies and the Editorial Policy Checklist.

Statistics

For all statistical analyses, confirm that the following items are present in the figure legend, table legend, main text, or Methods section.

n/a	Confirmed	
☐	☒	The exact sample size (n) for each experimental group/condition, given as a discrete number and unit of measurement
☐	☒	A statement on whether measurements were taken from distinct samples or whether the same sample was measured repeatedly
☐	☒	The statistical test(s) used AND whether they are one- or two-sided<br>*Only common tests should be described solely by name; describe more complex techniques in the Methods section.*
☐	☒	A description of all covariates tested
☐	☒	A description of any assumptions or corrections, such as tests of normality and adjustment for multiple comparisons
☐	☒	A full description of the statistical parameters including central tendency (e.g. means) or other basic estimates (e.g. regression coefficient) AND variation (e.g. standard deviation) or associated estimates of uncertainty (e.g. confidence intervals)
☐	☒	For null hypothesis testing, the test statistic (e.g. F, t, r) with confidence intervals, effect sizes, degrees of freedom and P value noted<br>*Give P values as exact values whenever suitable.*
☒	☐	For Bayesian analysis, information on the choice of priors and Markov chain Monte Carlo settings
☐	☒	For hierarchical and complex designs, identification of the appropriate level for tests and full reporting of outcomes
☐	☒	Estimates of effect sizes (e.g. Cohen's d, Pearson's r), indicating how they were calculated

Our web collection on statistics for biologists contains articles on many of the points above.

Software and code

Policy information about availability of computer code

Data collection	Bruker DRX-600, NeXion 300X spectrometer, FluoroMax-4 Spectrofluorometer, HORIBA FL-3, Edinburgh FLS1000,Shimadzu UV-1750 spectrophotometer
Data analysis	All statistical graph and fluorescent spectra were performed using Origin 8.5 (OriginLab Corporation, MA, USA). Quantum chemistry calculation was performed with Gaussian 0.9, revision D.01 (Gaussian Inc., Wallingford, CT, 2013). All the ball and sticks structures of were drawn through CYLview 20. The structure was interpreted and refined by SHELX-2014.

For manuscripts utilizing custom algorithms or software that are central to the research but not yet described in published literature, software must be made available to editors and reviewers. We strongly encourage code deposition in a community repository (e.g. GitHub). See the Nature Research guidelines for submitting code & software for further information.

Data

Policy information about availability of data

All manuscripts must include a data availability statement. This statement should provide the following information, where applicable:

- Accession codes, unique identifiers, or web links for publicly available datasets
- A list of figures that have associated raw data
- A description of any restrictions on data availability

The data supporting the findings of this study are available from the authors on reasonable request, see author contributions for specific data sets. Source data are provided as a source data file with this paper, underlying Fig. 1b, Fig. 2d, Fig. 3a-d,f-h, Fig. 4b,f,g, Fig. 6c, Supplementary Fig. 4-8, 12-16.The X-ray crystallographic coordinates for the structures reported in this article have been deposited at the Cambridge Crystallographic Data Centre (CCDC) under deposition numbers CCD 20477734 and 20477735. These data can be obtained free of charge from The Cambridge Crystallographic Data Centre via www.ccdc.cam.ac.uk/data_request/cif.

Field-specific reporting

Please select the one below that is the best fit for your research. If you are not sure, read the appropriate sections before making your selection.

☒ Life sciences ☐ Behavioural & social sciences ☐ Ecological, evolutionary & environmental sciences

For a reference copy of the document with all sections, see nature.com/documents/nr-reporting-summary-flat.pdf

Life sciences study design

All studies must disclose on these points even when the disclosure is negative.

Sample size	Each group was used three or four samples for in vivo and in vitro experiments. Statistics such as error bars, significance and p values can be derived from n≥3.
Data exclusions	No data were excluded.
Replication	All experimental were performed three times independently and all attempts replication were successful.
Randomization	All samples/organisms were divided into each groups randomly.
Blinding	The investigators were blinded to group allocation during data collection and/or analysis.

Reporting for specific materials, systems and methods

We require information from authors about some types of materials, experimental systems and methods used in many studies. Here, indicate whether each material, system or method listed is relevant to your study. If you are not sure if a list item applies to your research, read the appropriate section before selecting a response.

Materials & experimental systems

n/a	Involved in the study	
☒	☐	Antibodies
☒	☐	Eukaryotic cell lines
☒	☐	Palaeontology and archaeology
☐	☒	Animals and other organisms
☒	☐	Human research participants
☒	☐	Clinical data
☒	☐	Dual use research of concern

Methods

n/a	Involved in the study	
☒	☐	ChIP-seq
☒	☐	Flow cytometry
☒	☐	MRI-based neuroimaging

Animals and other organisms

Policy information about studies involving animals; ARRIVE guidelines recommended for reporting animal research

Laboratory animals	The balb/c female (nude) mice and C57BL/6 mice were purchased from SPF(Beijing) Biotechnology Co., Ltd. and they were raised in experimental animal barrier environment.
Wild animals	The study did not involve wild animals.
Field-collected samples	The study did not involve samples collected from the field.
Ethics oversight	All animal studies were performed according to the guidelines set by the Tianjin Committee of Use and Care of Laboratory Animals, and the overall project protocols were approved by the Animal Ethics Committee of Nankai University. The accreditation number of the laboratory is SYXK(Jin) 2019-0003 promulgated by Tianjin Science and Technology Commission.

Note that full information on the approval of the study protocol must also be provided in the manuscript.
